# Enterotoxigenic *Escherichia coli* Flagellin Inhibits TNF-Induced NF-κB Activation in Intestinal Epithelial Cells

**DOI:** 10.3390/pathogens6020018

**Published:** 2017-05-17

**Authors:** Gaochan Wang, Brian V. Geisbrecht, Christian Rueter, Philip R. Hardwidge

**Affiliations:** 1 Department of Diagnostic Medicine/Pathobiology, Kansas State University, Manhattan, KS 66506, USA; gaochan.wang@gmail.com; 2 Department of Biochemistry and Molecular Biophysics, Kansas State University, Manhattan, KS 66506, USA; GeisbrechtB@ksu.edu; 3 Institute of Infectiology, Center for Molecular Biology of Inflammation, University of Muenster, Muenster 48149, Germany; rueterchristian@hotmail.com

**Keywords:** ETEC, flagellin, NF-κB

## Abstract

Enterotoxigenic *Escherichia coli* (ETEC) causes childhood diarrhea in developing countries. ETEC strains produce the heat-labile enterotoxin (LT) and/or heat-stable enterotoxins (ST) and encode a diverse set of colonization factors used for adherence to intestinal epithelial cells. We previously found that ETEC secretes a heat-stable protein we designated as ETEC Secreted Factor (ESF) that inhibits the extent of NF-κB activation normally induced by tumor necrosis factor alpha (TNF). Here we fractionated ETEC supernatants using fast protein liquid chromatography (FPLC) and determined that ETEC flagellin was necessary and sufficient to protect IκBα from degradation in response to TNF stimulation. These data suggest a potentially novel mechanism by which ETEC may evade the host innate immune response by down-regulating NF-κB-dependent host responses.

## 1. Introduction

Enterotoxigenic *Escherichia coli* (ETEC) causes travelers’ diarrhea and diarrheal disease in children living in developing countries [1,2]. ETEC strains encode two main types of virulence factors—heat-labile and/or heat-stable toxins (LT and ST)—that cause watery diarrhea [3] and colonization factors (CFs) that mediate ETEC adherence to intestinal enterocytes [4]. In addition to causing diarrhea, LT enhances ETEC adherence to host cells by activating host signaling pathways, and inhibits antimicrobial peptide and cytokine (e.g., IL-8) production by disrupting nuclear factor-κB (NF-κB) signaling pathway activation [5].

Flagella play critical roles in ETEC virulence [6]. For example, in addition to their role in bacterial motility, flagella promote ETEC attachment to intestinal epithelial cells [7] and affect biofilm formation [8]. EtpA, an exoprotein adhesin, mediates ETEC adhesion between flagella and host cells [9].

NF-κB plays an important role in regulating inflammation and innate immune responses to microbial infections [10]. NF-κB is normally sequestered in the cytoplasm by the NF-κB inhibitor, IκBα. Upon TNF stimulation or microbial infection, the IκB kinase (IKK) complex is activated and phosphorylates IκBα which is then polyubiquitinated and degraded, resulting in nuclear translocation of NF-κB subunits [11]. The innate immune system recognizes pathogens through a diverse set of cellular pattern recognition receptors [12]. Pathogens have accordingly evolved mechanisms to subvert innate signaling pathways to promote their survival and transmission [13].

We previously reported that pre-incubation of HCT-8 cells with ETEC H10407 supernatant prevented TNF stimulation from inducing IκBα degradation and NF-κB activation [14]. We attributed this result to a heat-stable protein we designated as ETEC Secreted Factor (ESF). Here we fractionated ETEC supernatants and identified flagellin as necessary and sufficient for this phenomenon.

## 2. Results and Discussion

### 2.1. ETEC H10407 Secretes ESF into M9 Minimal Media

**Figure 1:**
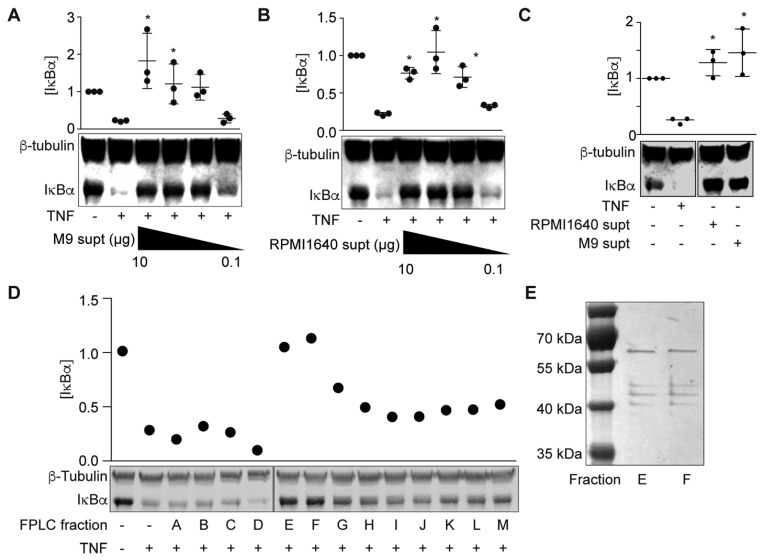
ESF fractionation and identification. (**A**) IκBα immunoreactivity after incubating HCT-8 cells with ETEC H10407-M9 supernatant (0.1–10 µg protein) and then stimulating the cells with TNF (20 ng/mL, 20 min) Asterisks indicate significantly different IκBα abundance as compared with the ‘TNF only’ lane; (**B**) IκBα immunoreactivity after incubating HCT-8 cells with ETEC H10407-M9 supernatant (0.1–10 µg protein) and then stimulating the cells with TNF (20 ng/mL, 20 min); (**C**) IκBα immunoreactivity after incubating HCT-8 cells with ETEC H10407-RPMI1640 and ETEC H10407-M9 supernatants (10 µg protein) without TNF; (**D**) IκBα immunoreactivity after incubating HCT-8 cells with ETEC H10407-M9 supernatant FPLC fractions for 1.5 h and then stimulating the cells with TNF (20 ng/mL, 20 min); (**E**) Sliver staining of FPLC fractions E and F on 10% SDS-PAGE.

We previously found that ETEC H10407 secretes a heat-stable protein (ETEC Secreted Factor or ESF) into RPMI 1640 medium that subsequently inhibits the ability of TNF to activate NF-κB signaling [14]. To facilitate identification of this protein using biochemical fractionation, we first determined whether this factor was also secreted into the ETEC supernatant when ETEC was grown in M9 minimal medium. We incubated HCT-8 cells with either cell-free ETEC-M9 or ETEC-RPMI 1640 supernatants for 1.5 h and then treated the cells with TNFα (20 ng/mL, 20 min). Pre-incubating HCT-8 cells with ETEC-M9 supernatant significantly inhibited IκBα degradation in response to TNFα stimulation (Figure 1A), similar to the results obtained from pretreating HCT-8 cells with ETEC-RPMI 1640 supernatant (Figure 1B; [14]). IκBα degradation was not observed in cells pre-incubated with only ETEC-RPMI 1640 or ETEC-M9 supernatants in the absence of TNF (Figure 1C). These data indicated that ETEC H10407 also secretes the ESF into M9 minimal medium.

Next, we used FPLC to fractionate ETEC-M9 supernatants and then assayed the fractions for their ability to block TNF-induced NF-κB activation. Two fractions (Figure 1D, fractions E and F) inhibited IκBα degradation in HCT-8 cells and silver staining data showed that these two fractions had a similar protein composition (Figure 1E). We excised these bands and identified the proteins using mass spectrometry (Table 1). We identified a major outer membrane lipoprotein, outer membrane protein A, the flagellar hook-associated protein FliD, and flagellin (FliC). *E. coli* K-12 strains do not encode the ESF [14]. The two outer membrane proteins are highly conserved between ETEC and *E. coli* K-12, but FliC and FliD are not (~50% identity). We therefore focused on FliC and FliD for subsequent biochemical assays.

### 2.2. ETEC Flagellin Blocks TNF-Induced IκBα Degradation

**Figure 2:**
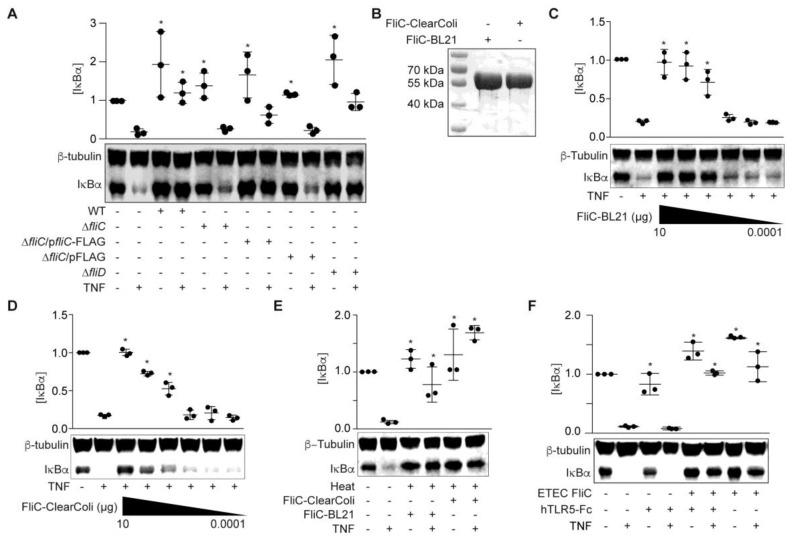
ETEC FliC blocks TNF-induced IκBα degradation. (**A**) IκBα immunoreactivity after incubating HCT-8 cells with WT and mutant ETEC supernatants (10 µg protein) and then stimulating the cells with TNF (20 ng/mL, 20 min); (**B**) Purified recombinant FliC resolved using 10% SDS-PAGE and Coomassie blue staining; (**C**) IκBα immunoreactivity after incubating HCT-8 cells with FliC-pET28a-BL21 (100 pg–10 µg) for 1.5 h followed by TNF stimulation (20 ng/mL, 20 min); (**D**) IκBα immunoreactivity after incubating HCT-8 cells with FliC-pET28a-ClearColi (100 pg–10 µg) for 1.5 h and then stimulating the cells with TNF (20 ng/mL, 20 min); (**E**) IκBα immunoreactivity after incubating HCT-8 cells with heated (100 °C, 20 min), recombinant FliC (1 µg) for 1.5 h followed by TNF stimulation (20 ng/mL, 20 min); (**F**) IκBα immunoreactivity after pretreating HCT-8 cells with 1.5 µg/mL hTRL5-Fc for 1 h, followed by FliC (100 ng/mL, 90 min) and TNF (20 ng/mL, 20 min).

To examine whether FliC and/or FliD protect HCT-8 cells from TNF-induced NF-κB activation, we generated ETEC H10407 *fliC* and *fliD* mutants and prepared cell-free supernatants from these mutant strains in RPMI 1640. ETEC Δ*fliC* supernatants failed to block TNF-induced IκBα degradation (Figure 2A).

Complementing ETEC Δ*fliC* with a *fliC* expression plasmid partially restored the protective phenotype (*p* = 0.1; Figure 2A). By contrast, the ETEC Δ*fliD* supernatant behaved more similarly to the WT ETEC supernatant, though these data did not reach statistical significance (*p* = 0.1; Figure 2A). We therefore concluded that *fliC* expression was necessary for the ESF phenotype. We next determined whether recombinant FliC is sufficient to account for the ESF phenotype. We expressed ETEC FliC in *E. coli* BL21 (DE3) and purified the recombinant protein using Ni-NTA chromatography (Figure 2B). FliC blocked IκBα degradation in response to TNF in a dose-dependent manner (Figure 2C).

To eliminate the potential impact of LPS contamination, we also expressed and purified FliC from *E. coli* ClearColi 21 (DE3). The ClearColi strain incorporates seven genetic deletions that inhibit carbohydrate modifications of the LPS molecule. Recombinant proteins expressed from ClearColi BL21 (DE3) do not need removal of contaminating LPS to avoid TLR4 activation [15]. Pre-incubating HCT-8 cells with FliC purified from ClearColi also protected IκBα from TNF-induced degradation in a dose-dependent manner (Figure 2D). The protective phenotype mediated by FliC was also heat-stable (Figure 2E), consistent with previous data [14].

To determine whether the FliC-mediated phenotype was abrogated by blocking Toll-like receptor 5 (TLR5) recognition, we first treated HCT-8 cells with the TLR5 antagonist hTLR5-Fc, followed by FliC and TNF. IκBα degradation in response to TNF stimulation was still inhibited by ETEC FliC, even after hTLR5-Fc pre-incubation (Figure 2F).

### 2.3. FliC Domain Mapping

The function of flagellin is broadly conserved among all flagellated bacteria. Nevertheless, outside the N- and C-terminal regions that comprise its intramolecular coiled-coil, the flagellin (FliC) protein itself exhibits localized sequence/structure variability between otherwise closely related bacteria. For example, whereas the central region of Salmonella FliC contains two distinct ~100 residue domains [16], sequence analyses suggest that ETEC FliC contains only a single, fused central domain consisting of residues 176–395. Importantly, residues within this central region of *E. coli* flagellin comprise variable H serotype-specific epitopes [6], suggesting that these sequences could impart strain-specific activities to the FliC protein.

**Figure 3:**
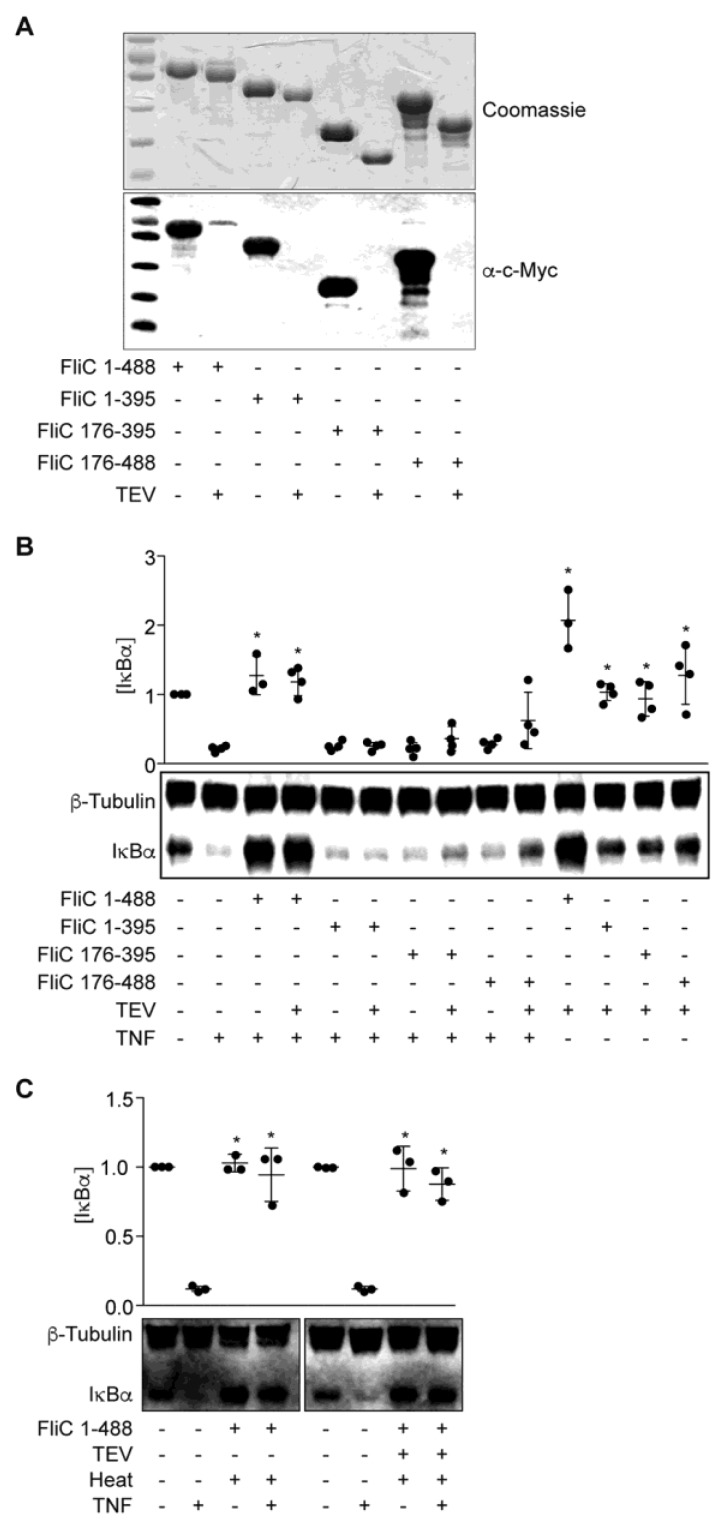
FliC truncations are inactive. (**A**) Purified FliC truncations +/− TEV protease treatment were resolved using 10% SDS-PAGE and analyzed by Coomassie blue staining (top) and Western blotting (bottom); (**B**) IκBα immunoreactivity after incubating HCT-8 cells with FliC truncations (1 µg) for 1.5 h followed by TNF stimulation (20 ng/mL, 20 min); (**C**) IκBα immunoreactivity after incubating HCT-8 cells with heated (100 °C, 20 min) FliC (1 µg) followed by TNF stimulation (20 ng/mL, 20 min).

To investigate if a specific FliC subdomain might account for the IκBα protective phenotype, we overexpressed and purified three truncated FliC proteins designated as FliC(1–395), FliC(176–395), and FliC(176–488) (Figure 3A). These proteins were expressed from pT7HMT to facilitate removal of the N-terminal His-tag using TEV protease. While the presence of a His-tag did not affect the activity of full-length FliC in blocking IκBα degradation in response to TNF (Figure 3B), none of the truncated FliC proteins were active. As a control, both tagged- and untagged-FliC were heat-stable, consistent with the heat-stability of the ESF we described previously (Figure 3C, [14]). 

### 2.4. Intracellular Expression of fliC Fails to Block IκBα Degradation in Response to TNFα

**Figure 4:**
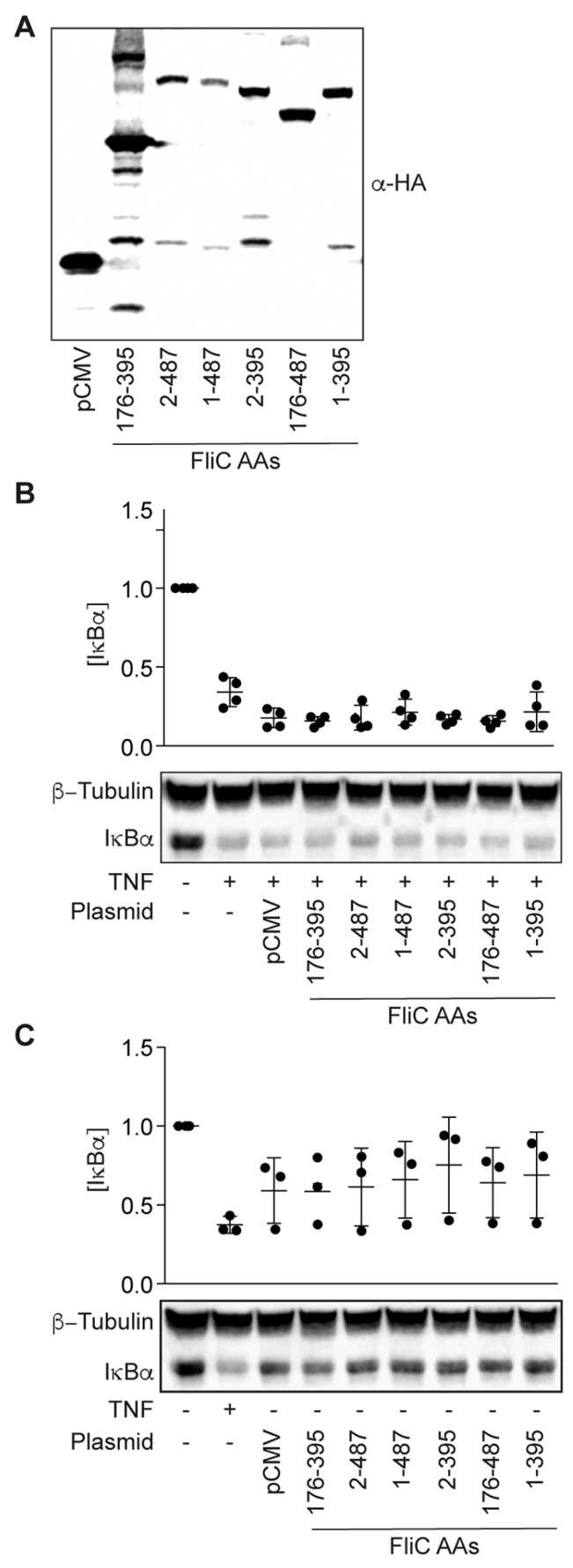
Intracellular expression of *fliC* fails to block IκBα degradation in response to TNFα. (**A**) Immunoreactivity of transfected FliC truncations expressed in HCT-8 cells 24 h post-transfection; (**B**) IκBα immunoreactivity in HCT-8 cells 24 h post-transfection of *fliC* truncations in the absence of TNF stimulation; (**C**) IκBα immunoreactivity in HCT-8 cells 24 h post-transfection of *fliC* truncations after TNF stimulation (20 ng/mL, 20 min).

We next sought to determine whether mammalian expression of FliC would be sufficient to block TNF-induced IκBα degradation. We expressed FliC fragments from a mammalian cell expression vector and transfected these plasmids into HCT-8 cells (Figure 4A). The expressed proteins were not toxic to HCT-8 cells (Figure 4B), but they were unable to block TNF-induced IκBα degradation (Figure 4C).

Here we identified ETEC FliC as necessary and sufficient to inhibit IκBα degradation in response to TNF stimulation (Figure 1 and Figure 2). Surprisingly, blocking TLR5 recognition of flagellin did not eliminate this phenotype, suggesting a potential TLR5-independent mechanism of action (Figure 2). We were unable to define the FliC subdomain responsible for this activity, despite truncating the protein into soluble fragments that comprised the variable central domain (Figure 3). We also observed that endogenous expression of FliC in mammalian cells did not reconstitute this phenotype (Figure 4), suggesting a potential extracellular target for FliC.

TNF-TNFR1-mediated NF-κB activation occurs on the cell surface and is associated with lipid rafts [17]. Cationic cell-penetrating peptides can disrupt TNF-mediated NF-κB signaling by inducing TNF receptor internalization in a clathrin-dependent manner [18].

The mechanism of FliC-mediated inhibition of IκBα degradation in response to TNF could be associated with TNFR internalization, as we had previously observed that blocking clathrin-dependent endocytosis affected the activity of the ETEC secreted factor (ESF; [14]). Future characterization of the phenotype we report here has the potential to provide insight to the development of anti-inflammatory compounds that target NF-κB, an approach that might prove efficacious in treating autoimmune disorders. These data also reinforce the notion that bacterial pathogens have evolved mechanisms to subvert the activation of host innate signaling pathways.

## 3. Materials and Methods

### 3.1. Reagents and Antibodies

Antibiotics were purchased from Fisher Scientific (Waltham, MA, USA). Restriction enzymes were purchased from New England BioLabs (Ipswich, MA, USA). TNFα was purchased from Cell Signaling Technology (Beverly, MA, USA). hTLR5-Fc was purchased from InvivoGen (San Diego, CA, USA). Antibodies were purchased from the following sources: HA, FLAG, and c-Myc (Sigma, St. Louis, MO, USA), His and tubulin (Santa Cruz, Dallas, TX, USA), IκBα (Cell Signaling Technology).

### 3.2. Bacterial Strains and Plasmids

Bacterial strains and plasmids are described in Table 2. ETEC mutants were derived from wild-type ETEC H10407 [19]. *E. coli* DH5α was used for routine molecular biological manipulations while *E. coli* BL21 (DE3) and ClearColi BL21 (DE3) were used for protein expression. Plasmids pKD3 and pKD46 were used to construct ETEC H10407 mutants. Plasmids pET28a and pT7HMT [20] were used to express recombinant proteins. All bacterial strains were grown aerobically in Luria-Bertani (LB) broth or on LB plates with antibiotics at 37 °C.

### 3.3. Cell Lines and Culture Conditions

The intestinal epithelial cell line HCT-8 was maintained at 37 °C, 5% CO_2_ in RPMI 1640 medium supplemented with 10% fetal bovine serum (FBS) and penicillin-streptomycin (100 U/mL). HCT-8 cells were seeded in 6-well plates at a concentration of 5 × 10^5^ cells/well. Media was replaced with fresh RPMI 1640 lacking both FBS and penicillin-streptomycin prior to experimentation.

### 3.4. ETEC Supernatant

ETEC H10407 culture supernatants were prepared in either RPMI 1640 or in M9 minimal medium (M9 salts, 2 mM MgSO_4_, 0.1 mM CaCl_2_, 0.4% glucose). Cell-free supernatants were concentrated by using Centricon Plus-70 Centrifugal Filters (EMD Millipore, Billerica, MA, USA) with a membrane Normal Molecular Weight Limit (NMWL) of 3 kDa and then precipitated using acetone. Protein pellets were collected by centrifugation at 15,000× *g*, 4 °C for 10 min, and resuspended in 0.15 M NaCl for fast protein liquid chromatography analysis.

### 3.5. Fast Protein Liquid Chromatography and Mass Spectrometry

ETEC H10407 supernatants were fractionated using a AKTA Fast Protein Liquid Chromatography (FPLC) system (GE Life Sciences, Marlborough, MA, USA) to facilitate molecular characterization of the ESF. Acetone-precipitated supernatants from M9-grown ETEC cultures were resuspended in 10 mL 0.15 M NaCl, clarified by 0.22 µm filtration, and applied at 4 mL/min to a Superdex S200 26/60 column (GE Life Sciences) that had previously been equilibrated in 20 mM Tris (pH 8.0), 200 mM NaCl. The ESF-containing eluent was collected from the column between 110–130 mL, and dialyzed against 4 L of 20 mM Tris (pH 8.0) in preparation for further chromatography. The ESF-containing sample was applied to a 1 mL Resource Q anion exchange column (GE Life Sciences). The column was washed with 20 mM Tris (pH 8.0) until the OD_280_ value reach baseline, and then the bound proteins were eluted with a gradient to 1 M NaCl in the same buffer. Fractions of 1 mL were collected and then screened for their ability to prevent IκBα degradation in response to TNF, separated by SDS-PAGE, and detected using Silver Staining (Thermo Scientific, Waltham, MA, USA). Proteins from active fractions were excised and digested in-gel with trypsin (Promega, Madison, WI, USA). Proteins were identified using mass spectrometry at the Mass Spectrometry & Analytical Proteomics Laboratory, University of Kansas.

### 3.6. Construction of ETEC H10407 Mutants

ETEC were generated using the Lambda Red Recombinase system [22]. PCR products containing chloramphenicol resistance cassettes were amplified from plasmid pKD3 using primers (Table 3) flanked with homologous upstream and downstream gene sequences. PCR products were transformed into ETEC H10407 carrying the pKD46 plasmid via electroporation. Potential mutants were screened on LB plates containing chloramphenicol. All mutants were confirmed using DNA sequencing. ETEC H10407 *fliC* was also expressed from pFLAG-CTC for complementation studies.

### 3.7. Recombinant Protein Expression and Purification

Recombinant protein expression was induced with 1 mM IPTG, at 37 °C for 5 h. Recombinant proteins were purified by using nickel-affinity chromatography and subsequently dialyzed into PBS. Purified proteins were analyzed on 12% SDS-PAGE and concentrations were quantified using the Bradford method.

### 3.8. Transfection

HCT-8 cells were seeded in 6-well plates at a concentration of 2 × 10^5^ cells/well, grown to 70–80% confluence, and transfected with 2.5 µg plasmid DNA using Lipofectamine 3000 (Life Technology, Carlsbad, CA, USA). Transfected cells were stimulated with TNFα (20 ng/mL) at 12–48 h post-transfection.

### 3.9. Immunoblotting

HCT-8 cell pellets were resuspended in 10.0 mM HEPES, 1.5 mM MgCl_2_, 10.0 mM KCl, 0.5 mM DTT, 0.05% NP-40 containing protease inhibitor cocktails (Thermo Scientific) and incubated on ice for 30 min. Lysates were centrifuged (10,000× *g*, 4 °C, 10 min) and the supernatant was collected. Immunoblotting was carried out as previously described [14] by separating proteins using 10% SDS-PAGE and then transferring the proteins to nitrocellulose membranes. Membranes were blocked in Odyssey blocking buffer (Li-Cor, Lincoln, NE, USA) at room temperature for 1 h, and then incubated with appropriate primary and secondary primary antibodies. Immunoblots were developed using the Odyssey infrared imaging system (Li-Cor). Tubulin abundance was used to normalize IκBα abundance.

### 3.10. Statistical Analysis

For all quantitative data, tubulin immunoblotting was used to normalize IκBα abundance. The data represent at least 3 independent experiments and were analyzed using one-way ANOVA with the Dunnett’s multiple comparisons test. Asterisks indicate significantly different IκBα abundance as compared with the ‘TNF only’ lane. Statistical differences were evaluated using one-way ANOVA with the Dunnett’s multiple comparisons test. *p*-values < 0.05 were considered significant.

## Figures and Tables

**Table 1:** Mass spectrometry results.

Protein Candidates	Sequence Coverage	Identity to *E. coli* MG1655	GenBank Accession #
Major outer membrane lipoprotein	49% over 78 AAs	100%	CBJ00536.1
Outer membrane protein A	26% over 346 AAs	99%	CBJ01214.1
Flagellar hook-associated protein 2 (FliD)	44% over 470 AAs	50%	CBJ01536.1
Flagellin (FliC)	71% over 487 AAs	52%	CBJ01535.1

**Table 2:** Strains and plasmids used in this study.

Strain or Plasmid	Description	Source or Reference
*Strains*		
ETEC H10407	O78:H11, CFA/I, LT^+^ and ST^+^	[19]
*E. coli* DH5α	Cloning strain	New England BioLabs
*E. coli* BL21 (DE3)	Protein overexpression strain	Novagen
ClearColi BL21 (DE3)	Protein overexpression	Lucigen
ETEC Δ*fliC*	ETEC H10407 *fliC* mutant	This study
ETEC Δ*fliD*	ETEC H10407 *fliD* mutant	This study
ETEC Δ*fliC*/pFliC-FLAG	ETEC H10407 Δ*fliC* complemented with *fliC*	This study
*Plasmids*		
pFLAG-CTC	FLAG-tagged protein expression	Sigma
pET28a	His_6_ fusion protein expression	Novagen
pT7HMT	His_6_ fusion protein expression with TEV site	[20]
pKD3	Template for mutagenic PCR products	[21]
PKD46	Lambda Red mediated mutagenesis	[21]
pCMV	Mammalian expression vector with HA-tag	[22]
pET28a-FliC	FliC in pET28a	This study
pT7HMT-FliC	FliC in pT7HMT	This study
pT7HMT-FliC (176–395)	FliC (176–395) in pT7HMT	This study
pT7HMT-FliC (1–395)	FliC (1–395) in pT7HMT	This study
pT7HMT-FliC (176–488)	FliC (176–488) in pT7HMT	This study
pCMV-FliC (1–487)	FliC (1–487) in pCMV	This study
pCMV-FliC (176–395)	FliC (176–395) in pCMV	This study
pCMV-FliC (1–395)	FliC (1–395) in pCMV	This study
pCMV-FliC (176–487)	FliC (176–487) in pCMV	This study
pCMV-FliC (2–395)	FliC (2–395) in pCMV	This study
pCMV-FliC (2–487)	FliC (2–487) in pCMV	This study

**Table 3:** Oligonucleotides used in this study.

Primer	Purpose	Sequence (5′-3′)
PRH-3427	Delete ETEC H10407 *fliD*	A_2_T_2_GC_2_GATA_2_C_3_GCT_2_ATCTACTGT_3_GCA_2_TC
		A_4_G_2_A_2_T_2_AG_2_TGTGTA2CTG_2_AGCTGCT_2_C
PRH-3428	Delete ETEC H10407 *fliD*	T_2_GTGCATAG_2_CT_4_GAGC_2_GCTCGCG_2_TATAC
		ATGCTGAC_2_TC_2_GTGA_2_TG_3_A_2_T_2_AGC_2_ATG_2_TC_2_
PRH-3429	Verify *fliD* deletion	TCTCTC_2_TGT_6_CT_2_A_2_CG_2_CT
PRH-3430	Verify *fliD* deletion	GCTGAT_2_GT_2_GTC_2_TGCATA_3_CA
PRH-3431	Delete ETEC H10407 *fliC*	CGTG_3_CA_2_CAGC_3_A_2_TA_2_CATCA_2_GT_2_GTA_2_T_2_GA
		TA_2_G_2_A_4_GATCGTGTAG_2_CTG_2_AGCTGCT_2_C
PRH-3432	Delete ETEC H10407 *fliC*	GCG_3_CAGA_6_C_4_GC_2_G_2_TG2CG_5_T_2_GAGCGA
		TA_2_GTGTA_4_TG_3_A_2_T_2_AGC_2_ATG_2_TC_2_
PRH-3433	Verify *fliC* deletion	ATGATGCGCAGAGTAGAGT_2_GTAT
PRH-3434	Verify *fliC* deletion	ATGAT_2_ATC_2_GT_3_CTGCAG_3_T_2_
PRH-3619	Clone *fliC* pCMV-XhoI	TAC_2_GCTCGAGATG_2_CACA_2_GTCAT_2_A_2_TA
PRH-3620	Clone *fliC* pCMV-NotI	ATA_2_GA_2_TGCG_2_C_2_GCACGCAGCAGAGACAGTA
PRH-3681	Clone *fliC* pET28a-Nde I	GGA_2_T_2_C_2_ATATG_2_CACA_2_GTCAT_2_A_2_TACA
PRH-3682	Clone *fliC* pET28a-XhoI	TAC_2_GCTCGAGACGCAGCAGAGACAGTA
PRH-3684	Clone *fliC* pFLAG-CTC-XhoI	TAC_2_GCTCGAG_2_CACA_2_GTCAT_2_A_2_TA
PRH-3685	Clone *fliC* pFLAG-CTC-BglII	G_2_A_2_GATCTACGCAGCAGAGACAGTA
PRH-3788	Clone *fliC* 176-395 pCMV-XhoI	TATAT_2_ACTCGAG_2_ATG_2_CGCGCAGA_3_GCA_2_
PRH-3789	Clone *fliC* 176-395 pCMV-NotI	ATA_2_GA_2_TGCG_2_C_2_GCT_2_GCA_2_CGAT_4_
PRH-3543	Clone *fliC* pT7HMT-BamHI	TACGCG_2_ATC_2_ATG_2_CACA_2_GTCAT_2_A_2_TACA_2_
PRH-3844	Clone *fliC* pT7HMT-NotI	ATA_2_GAT_2_GCG_2_C_2_GCT_2_A_2_CGCAGCAGAGA
PRH-3845	Clone *fliC* 176-395 pT7HMT-BamHI	TACGCG_2_ATC_2_GATG_2_CGCGCAGA_3_
PRH-3851	Clone *fliC* 176-395 pT7HMT-NotI	ATA_2_GAT_2_GCGGC_2_GCTCAT_2_GCA_2_CGAT_4_
PRH-3971	Clone *fliC* pCMV-XhoI	TAC_2_GCTCGAG_2_CACA_2_GTCAT_2_A_2_TACA_3_CAGC_2_

## Abbreviations

ETEC	enterotoxigenic *Escherichia coli*
LT	heat-labile enterotoxin
NF-κB	nuclear factor-κB
ST	heat-stable enterotoxin
TNFα	tumor necrosis factor α
